# Maternity and Mothering

**Published:** 1912-02-24

**Authors:** 


### MATERNITY AND MOTHERING.

(Inquiries and Correspondence are invited)

### The Up-to-date Treatment of Ringworm.

There are few childish ailments which last so long or cause so much distress to parents and guardians as ringworm. The children themselves do not, it is true, suffer any bodily discomfort from it; but the necessary interruption of their educational life for long periods is a serious disadvantage to them, though they may not appreciate the fact themselves.

The name of the disease is misleading, inasmuch as it is not due to a "worm," or indeed to any animal parasite. The true cause is the growth of a microscopic fungus in and around the roots of the hair. Though the hair itself dies, the actual root is not killed; so that if the fungus can be got rid of, the hair will in time grow again as luxuriantly as ever. The idea that any bald patch on the head of a child is evidence of ringworm is an error; ringworm patches are often not completely bald, though the hair may be very much thinned; and there are other quite different causes of round bald areas. The diagnosis of ringworm, indeed, can only be made with absolute certainty by a medical man, although experienced school nurses can often give an opinion which will be right in the majority of cases.

The treatment of ringworm aims at the removal of all affected hairs, the eradication of the fungus, and the prevention of any spread of the disease to other patients. For the latter object it is important that the cap, hat, hairbrush, comb, flannel, sponge, and so on should be used by the patient alone, and not touched by any other child. Another indispensable regulation is that the child shall wear a paper cap, or a paper lining to his hat, which can be burnt at the end of the day. Every effort to prevent him from scratching his head is also to be made, though this is of course difficult. These measures, it will be seen, are in the hands of the mother or nurse, and much is gained if they are strictly and carefully carried out.

For the actual cure of the ringworm various ointments and applications have been advised; as will be presently seen, the tendency in recent years has been to rely upon newer and quite different ways of securing the eradication of the fungus. To pluck out as many of the diseased hairs as possible with a fine forceps is an excellent way to start; the affected hairs are loose, and come away without causing pain. Then an antiseptic lotion, preferably one containing no water and nothing of a fatty nature, should be applied. Ten to twenty grains of salicylic acid dissolved in an ounce of ether is an old favourite. Tincture of iodine has also been used, and chrysarobin has been the sheet anchor of two generations of practitioners. In any case the selected remedy must be used perseveringly and regularly until the doctor pronounces the head to be free of the disease. This takes at least six weeks under drug treatment; more often three or four months; and not at all uncommonly the best part of a year, or even more than that. This chronic course and refractory character of ringworm were long the despair of patients, parents, and the medical profession. Consequently the discovery a few years ago that the judicious use of *x*-rays could be relied upon to reduce the duration of the disease from months to weeks, and that this could be effected in one application, or in two or three at most, rendered the resort to this method very widespread.

The disastrous results of prolonged exposure to *x*-rays have for long been before the public, owing to the dreadful fate which overtook some of the early pioneers of this form of treatment. In dealing with ringworm, however, the short duration of the treatment and the precautions with which it is administered preclude the possibility of any development of cancer or other form of malignant disease. Nor has any case been recorded, so far as we are aware, of injury to the brain from *x*-rays thus used, though this possibility was suggested and discussed when the London County Council two or three years ago decided to adopt the treatment for children in the elementary schools. The protection of the patient from such after-effects is gained by the use of an invention of a celebrated Parisian specialist, Dr. Sabouraud. A pastille is exposed to the rays at the same time as the affected region of the scalp; when the pastille has changed colour to a certain tint the dose of *x*-rays given is sufficient, and the treatment is then suspended.

Now there is no doubt that this method does cure ringworm with certainty and with a rapidity such as no previous treatment could ensure. Unfortunately, experience has shown that a certain number of the children thus treated become permanently bald—i.e., that the rays not only kill the microscopic fungus which is the cause of ringworm, but kill the hair roots also. Some *x*-ray experts still contend that if due care and sufficient precautions are taken this result need not and will not ensue. But it is admitted by the great majority that no guarantee can be given that the hair will grow again after the diseased hair has been shed. Authorities variously estimate the proportion of patients in whom this failure to regenerate the hair after the disease is cured has followed the *x*-ray treatment. Two per cent. is admitted by several radiologists to be a fair estimate, and some other experts believe the percentage to be greater even than this.

There is, however, another form of physical therapy which has become popular with some dermatologists since the *x*-rays got into bad repute for causing permanent baldness. This is the ionic method, which has been described recently in detail by Dr. Riddell.* If its claims prove well founded the *x*-rays as a method of treating ringworm will be unjustifiable in future.

* *Glasgow Medical Journal*, February 1912.

